# HoxA and HoxD expression in a variety of vertebrate body plan features reveals an ancient origin for the distal Hox program

**DOI:** 10.1186/2041-9139-5-44

**Published:** 2014-11-19

**Authors:** Sophie Archambeault, Julia Ann Taylor, Karen D Crow

**Affiliations:** Department of Biology, San Francisco State University, 1600 Holloway Ave, San Francisco, CA 94132 USA

**Keywords:** Hox expression, reverse collinear, HoxA, HoxD, vertebrate development, morphological diversity

## Abstract

**Background:**

Hox genes are master regulatory genes that specify positional identities during axial development in animals. Discoveries regarding their concerted expression patterns have commanded intense interest due to their complex regulation and specification of body plan features in jawed vertebrates. For example, the posterior HoxD genes switch to an inverted collinear expression pattern in the mouse autopod where *HoxD13* switches from a more restricted to a less restricted domain relative to its neighboring gene on the cluster. We refer to this program as the ‘distal phase’ (DP) expression pattern because it occurs in distal regions of paired fins and limbs, and is regulated independently by elements in the 5′ region upstream of the HoxD cluster. However, few taxa have been evaluated with respect to this pattern, and most studies have focused on pectoral fin morphogenesis, which occurs relatively early in development.

**Results:**

Here, we demonstrate for the first time that the DP expression pattern occurs with the posterior HoxA genes, and is therefore not solely associated with the HoxD gene cluster. Further, DP Hox expression is not confined to paired fins and limbs, but occurs in a variety of body plan features, including paddlefish barbels - sensory adornments that develop from the first mandibular arch (the former ‘Hox-free zone), and the vent (a medial structure that is analogous to a urethra). We found DP expression of *HoxD13 and HoxD12* in the paddlefish barbel; and we present the first evidence for DP expression of the HoxA genes in the hindgut and vent of three ray-finned fishes. The HoxA DP expression pattern is predicted by the recent finding of a shared 5′ regulatory architecture in both the HoxA and HoxD clusters, but has not been previously observed in any body plan feature.

**Conclusions:**

The Hox DP expression pattern appears to be an ancient module that has been co-opted in a variety of structures adorning the vertebrate bauplan. This module provides a shared genetic program that implies deep homology of a variety of distally elongated structures that has played a significant role in the evolution of morphological diversity in vertebrates

**Electronic supplementary material:**

The online version of this article (doi:10.1186/2041-9139-5-44) contains supplementary material, which is available to authorized users.

## Background

Hox genes are conserved, developmental regulatory genes that occur in all bilaterians. They are arranged in clusters and play a key role in animal development by specifying positional identities through nested and overlapping expression domains. This is referred to as ‘the Hox code’ and is accomplished through spatial, temporal, and quantitative collinearity. Collinear Hox expression, which has been described as ‘a spectacular phenomenon that has excited life scientists since its discovery in 1978′ [1], means that the order in which the genes occur on the chromosome is the order in which they are expressed in the organism [2, 3], as defined by their anteriormost expression domain. During early animal development, collinear Hox expression sets up anterior-posterior patterning where the genes on the 3′ end of the cluster are expressed earlier in anterior domains, followed by the progressive and more posterior expression of genes located toward the 5′ end. The HoxA and HoxD genes are deployed in a similar manner during limb development to pattern the proximal limb, including the arm and forearm. An important distinction of collinear expression is that the 5′ genes have restricted expression domains relative to their 3′ neighbors on the cluster. This pattern of collinear Hox expression is sometimes called the ‘general Hox strategy,’ in part, because an alternative, inverted expression pattern has been observed with the 5′ (posterior) HoxD genes in distal regions of vertebrate fins and limbs. The latter is associated with a switch in cis-regulatory regions [4] from the telomeric side of the cluster (3′) to the centromeric side (5′) [5, 6], and is manifest as a broader expression domain of the (5′) gene relative to its 3′ neighbor on the cluster. This unique expression pattern has been interchangeably referred to as ‘inverted’, ‘inverse’, ‘late-phase’, ‘autopodial-like’, or ‘reverse-collinear’ expression. Here, we refer to this pattern as ‘distal phase’ (DP) expression because it is associated with specification of distal structures and is regulated independently, but does not always follow an earlier collinear phase and is not solely associated with fins and limbs. To clarify, both proximal and distal expression patterns meet the definition of collinearity, but are differentiated with respect to their regulatory regions and the relative expression patterns of the genes in closest proximity to the active regulatory region.

Distal phase expression was first observed for of the posterior HoxD genes in distal regions of paired limbs, during later stages of development, and has commanded intense interest due to its complex regulation and specification of novel structures, such as the thumb during digit development [7, 8]. Early comparative analyses of Hox gene expression in zebrafish suggested that this expression pattern was an evolutionary novelty in tetrapods, garnering a substantial amount of attention [9, 10]. However, it was later discovered that the posterior HoxD genes exhibit the DP expression pattern in the distal region of non-tetrapod appendages, including the pectoral fins of the paddlefish (a basal ray-finned fish) [11] and the pectoral and pelvic fins of the catshark [12], indicating that DP HoxD expression arose in the common ancestor of jawed vertebrates. This unique expression pattern has been observed only with the posterior genes of the HoxD cluster during distal fin and limb development, with a characteristic switch from proximal/early (collinear) to distal/late-phase/reverse collinear expression. Shubin, Tabin and Carroll [13] defined the pattern as having four important features in the tetrapod autopod: (1) it occurs in distal domains, (2) it occurs when bony elements are being specified, (3) it is regulated independently from early collinear expression, and (4) it results in broader expression domains of the 5′ gene *(HoxD13*), which extends more anteriorly than its 3′ neighboring gene on the cluster (*HoxD12*).

Multiple authors have focused on criterion (4) as a defining feature of DP HoxD expression in fins and limbs [11, 12, 14] when *HoxD13* expression switches from posteriorly restricted to more anterior and broad expression in distal regions of vertebrate appendages. Therefore, demonstration of the DP expression pattern has hinged on demonstrating the broader expression domain of *HoxD13* relative to *HoxD12*. For example, the HoxD genes exhibit the general Hox strategy with collinear expression and the more posterior genes exhibit progressively smaller expression domains during early development of limbs. However, there is a subsequent switch to DP expression of the posterior HoxD genes [9–12] in the distal regions of the autopod during digit formation and in paired fins [5, 7, 8], for example, when *HoxD13* is expressed broadly in all five digits in the mouse and human autopod while *HoxD12* is restricted to digits 2 to 5 (that is, excluded from digit 1) resulting in specification of the thumb [1, 7, 15].

More recent work has focused on criterion (3), or identification of independent cis-regulatory regions associated with HoxD collinear, and reverse collinear expression patterns. Collinear expression of HoxD genes in the proximal region of the mouse limb is regulated primarily by cis-regulatory elements located in a gene desert on the telomeric (towards the 3′) side of the cluster [5]; however, specific enhancers have yet to be identified. Subsequently, there is a switch to DP expression in the distal portion of the mouse limb that is associated with a conformational change in chromatin structure involving enhancers on the centromeric (5′) end of the cluster [5, 16]. More is known about the centromeric regulatory archipelago [8], with multiple elements identified that are able to drive distal expression in mice and zebrafish [5, 7, 8, 17]. Several of these exhibit high conservation among jawed vertebrates (for example, elements I, III and CsB are conserved in human, mouse, chick, frog, zebrafish, pufferfish and skate) [14, 17, 18]. The CsB element from the skate and the zebrafish has been evaluated in transgenic mice, resulting in distal expression and supporting the notion that regulation of late phase DP HoxD is conserved [17]. However, three elements are tetrapod-specific, reflective of the morphological disparity between the autopod and paired fins. Current models explain regulation of DP expression as having two components: 1) proximity of the gene to a 5′ cis-regulatory looped complex and 2) gene specific promoter affinities [7, 8]. As a result, the broader expression domain of the 5′ gene, in closest proximity to the centromeric regulatory landscape, has been explained by increased transcriptional efficiency (that is, quantitatively more transcript), and deviations from strictly decreasing transcription (for example, identical expression domains of *HoxD12* and *HoxD11* in the mouse autopod) is explained by enhancer affinity for gene specific promoters [7].

The posterior HoxA genes have not been reported to exhibit DP expression, but a ‘late phase’ expression has been described in tetrapod limbs (reviewed in [14, 19]) that is characterized by collinear but non-overlapping expression, which has been attributed to the exclusion of *HoxA11* by *HoxA13* expression in the distal autopod [20–24]. Interestingly, the ‘early phase’ of HoxA expression in paddlefish pectoral fins mimics the ‘late phase’ pattern in tetrapod limbs (see [25]), but this similarity has not previously been recognized in the literature. Expression in the catshark has not been evaluated with respect to these genes. Several studies have identified cis-regulatory landscapes located on both sides (5′ and 3′) of the HoxA cluster that exhibit similar conformational properties as the HoxD cluster in zebrafish [26] and mice [26, 27], suggesting that the 5′ regulatory landscape may have been present before the duplication of the HoxA and HoxD clusters [26–28]. This shared regulatory landscape predicts the possibility of HoxA DP expression, but it has not been observed in fins or limbs.

As the most diverse group of vertebrates, the ray-finned fishes exhibit a remarkable array of body plan features, including fin modifications and distally elongated structures such as vents, barbels, exaggerated rostrums (for example, paddlefish), and dermal appendages (for example, seadragons). Because these features do not have clear homologs in humans, their evolution and development are not well characterized. However, they contribute to the morphological diversity observed in a variety of lineages, and their novelty provides a unique opportunity for elucidating general principles of morphological evolution. Looking beyond the development of fins and limbs, we demonstrate that the DP expression pattern of both the HoxA and HoxD genes are associated with the development of a variety of body plan features in various lineages of ray-finned fishes. Our hypothesis is that the Hox ‘limb-building tool kit’ is a module that is deployed more universally than previously recognized, in a variety of distally elongated vertebrate structures.

## Methods

### Embryos and staging

Paddlefish embryos were obtained from the Tishomingo National Fish Hatchery. Yolk-sac larvae were staged according to Ballard and Needham [29] and Bemis and Grande [30] and sampled from hatching (stage 37) to the onset of feeding (stage 46). For the larval to juvenile stages of development (stages 47 to 53), samples were characterized for consistent developmental markers with replication of multiple individuals per stage. Zebrafish and goby work was conducted in accordance with protocols approved by the Institutional Animal Care and Use Committee of San Francisco State University. These embryos were obtained from broodstock reared according to the protocols of Westerfield [31] and Archambeault *et al.*[32] respectively. Zebrafish were staged according to Kimmel and Ballard [33], and gobies according to Archambeault *et al.*[32]. Zebrafish embryos were dechorionated prior to fixation, whereas goby embryos were dechorionated after fixation.

### Transcriptome analysis

Paddlefish larvae (stages. 52 to 53) were preserved in RNAlater and then dissected to include the region anterior to the upper lip (that is the rostrum and paired barbels). RNA was extracted using Ambion’s RNAqeous kit with DNAse removal. After library construction and Illumina™ sequencing of the transcriptome (UC Davis Genome Sequencing Center, Davis, CA, USA), the analysis produced 10,974,352 80-bp nonpaired end reads that assembled into 9.3 Mb of expressed sequence. Based on the average transcriptome size of similar species, we estimate partial coverage of roughly two-thirds of all expressed genes. The known paddlefish *HoxA*α and *HoxA*β sequences, and the *HoxD11, HoxD12 and HoxD13* genes from zebrafish were aligned to the paddlefish rostrum transcriptome reads using TBLASTN and generated significant results (for example, *HoxA11*α in Additional file 1: Figure S1). We used reciprocal BLAST (both E <10^-6^) analyses to identify putative orthologs of specific Hox paralogs represented in the transcriptome.

### Whole-mount *in situ*hybridization

Probes were constructed for the two posteriormost, neighboring genes on the HoxA (*HoxA11 and HoxA13*) and the HoxD (*HoxD13 and HoxD12*) clusters. Individual Hox genes were PCR amplified using paralog-specific primers (Table 1), cloned using the pGEM™-T Vector System II (Promega Corporation, Madison, WI, USA), and linearized using NcoI, SpeI or SphI (Promega Corporation). Digoxigenin-labelled RNA probes were synthesized according to Wilkinson [34]. Paddlefish *in situ* hybridization was performed following the protocols of Moens [35] and Thisse and Thisse [36] whereas zebrafish and goby *in situ* hybridizations were performed as described by Wilkinson [34]. Proteinase K digestion, hybridization temperature, probe concentration, blocking conditions and BCIP/NBT staining duration were empirically optimized for each taxon and gene. Following colorization, staining was intensified through methanol washes, and embryos were re-fixed in 4% PFA and photographed in glycerol.

**Table 1:** **Summary of Hox genes implicated in paddlefish rostrum and vent development**

			Evidence for expression		
Gene	BAC construct	Paralog	Rostrum transcriptome	Barbel ***in situ***	Vent ***in situ***
HoxA13	352P4	α	**-**	**-**	**√**
	370N10	β	**-**		
HoxA11	352P4	α	**√**	**√**	**√**
	370N10	β	**-**		
HoxD13	231C24	α	**√**	**√**	**√**
	249G23	β	**√**	**-**	
HoxD12	231C24	α	**√**	**√**	**√**
	249G23	β	**-**		
HoxD11	231C24	α	**√**		
	249G23	β	**-**		

Constructs refer to paddlefish BAC clones curated and accessioned by Chris Amemiya, Benaroya Research Institute [37]. Blank indicates expression has not been evaluated and a dash indicates we looked, but there are no data that support expression.

### Hox paralogs and probe specificity

All three ray-finned fishes included in this study have duplicated genomes. The paddlefish lineage experienced a whole genome duplication (WGD) approximately 42 million years ago [37], whereas the zebrafish and goby share a WGD that occurred in the stem lineage of teleosts [37] approximately 200 million years earlier. As such, the coding regions of the paddlefish Hox paralogs, which diverged relatively recently, exhibit little sequence divergence (ranging from 3 to 11%, see [37]). For thoroughness, we constructed paralog-specific probes for the *HoxD13* paralogs in the paddlefish and *HoxA11* and *HoxA13* paralogs in zebrafish and goby (the relevant HoxD gene copies have been lost in these taxa). Both paddlefish HoxD13 paralogs were implicated in the rostrum transcriptome, and fortuitously, these genes exhibit the highest sequence divergence among all HoxD paralogs, and therefore exhibit the highest probability of probe binding specificity. We constructed paralog-specific probes from within exon one (with 11% sequence divergence between probes) and an additional set of probes from the 5′ UTR, (with 32% sequence divergence), spanning 500 bp upstream of the start codon [see Additional file 2: Figure S2]. *HoxA11*, *A13*, and *D12* probes were specific to the alpha paralog, spanning 500 to 600 bp within exon 1 [see Additional file 2: Figure S2, Additional file 3: Table S1]. Multiple *in situ* hybridizations were conducted on paddlefish embryos with two *HoxD13α* and *β* specific probes from different regions. Previous work indicated that the alpha paralogs may been evolving neutrally, whereas the beta paralogs are under selection, which was interpreted as evidence for transcriptional inactivity of the entire HoxAα and HoxDα clusters [37]. However, our transcriptomic data indicate that the alpha paralogs are transcriptionally active in paddlefish.

The zebrafish and goby HoxA paralogs are older and more divergent than paddlefish, and functional divergence between *HoxA13* paralogs in zebrafish has been demonstrated [38]. We conducted paralog specific *in situ* hybridizations for *HoxA11* and *HoxA13* in zebrafish and goby. The zebrafish lineage has only one copy of the HoxD cluster, and therefore, no HoxDb paralogs exist. Percomorphs (represented here by the goby) have lost both copies of the *HoxD13* and one copy of the *HoxD12* gene [39], making comparative expression data for these genes irrelevant.

## Results and discussion

### Hox genes are expressed in a derivative of pharyngeal arch I, the former ‘Hox-free zone’

Initially, we hypothesized that Hox genes may play a role in the development of the paddlefish rostrum - an extreme, distally elongated feature that serves as an antenna for prey detection in this highly derived, basal actinopterygian. However, Hox genes are not known to be expressed in domains that occur anterior to pharyngeal arch II (the hyoid arch, [40]), and their absence is critical for proper jaw development [41]. To investigate any potential role for Hox expression in the development of the paddlefish rostrum, we sequenced the transcriptome of the rostrum anlagen from four individuals at stage 52 (n = 2, 26 dpf, onset of rostrum development), and stage 53 (n = 2, 28 dpf, early elongation) and found evidence for expression of several Hox genes. We queried our transcriptome reads with full sequences of the duplicated HoxA and HoxD paddlefish clusters [42] using reciprocal BLASTN (E <10^-6^) to establish orthology and paralogy of specific Hox genes within the transcriptome [see Additional file 1: Figure S1].

These data provide clear evidence for expression of several posterior genes from the ‘Hox limb-building tool kit’ including *HoxA11*, *HoxD11, HoxD12*, and *HoxD13* in the paddlefish rostral domain (Table 1, see section on Hox paralogs for evidence of specific paralog expression). To our knowledge, this is the first evidence for Hox expression in a derivative of pharyngeal arch I, which has been referred to as the ‘Hox-free zone’ [40]. To evaluate specific expression domains and relative patterns of multiple genes, we performed *in situ* hybridization on the paddlefish rostrum for both whole mount embryos and histological sections. Interestingly, we found no evidence for Hox expression in the rostrum, but rather verified that the Hox genes indicated in the transcriptome are expressed in the paddlefish barbel.

**Figure 1:**
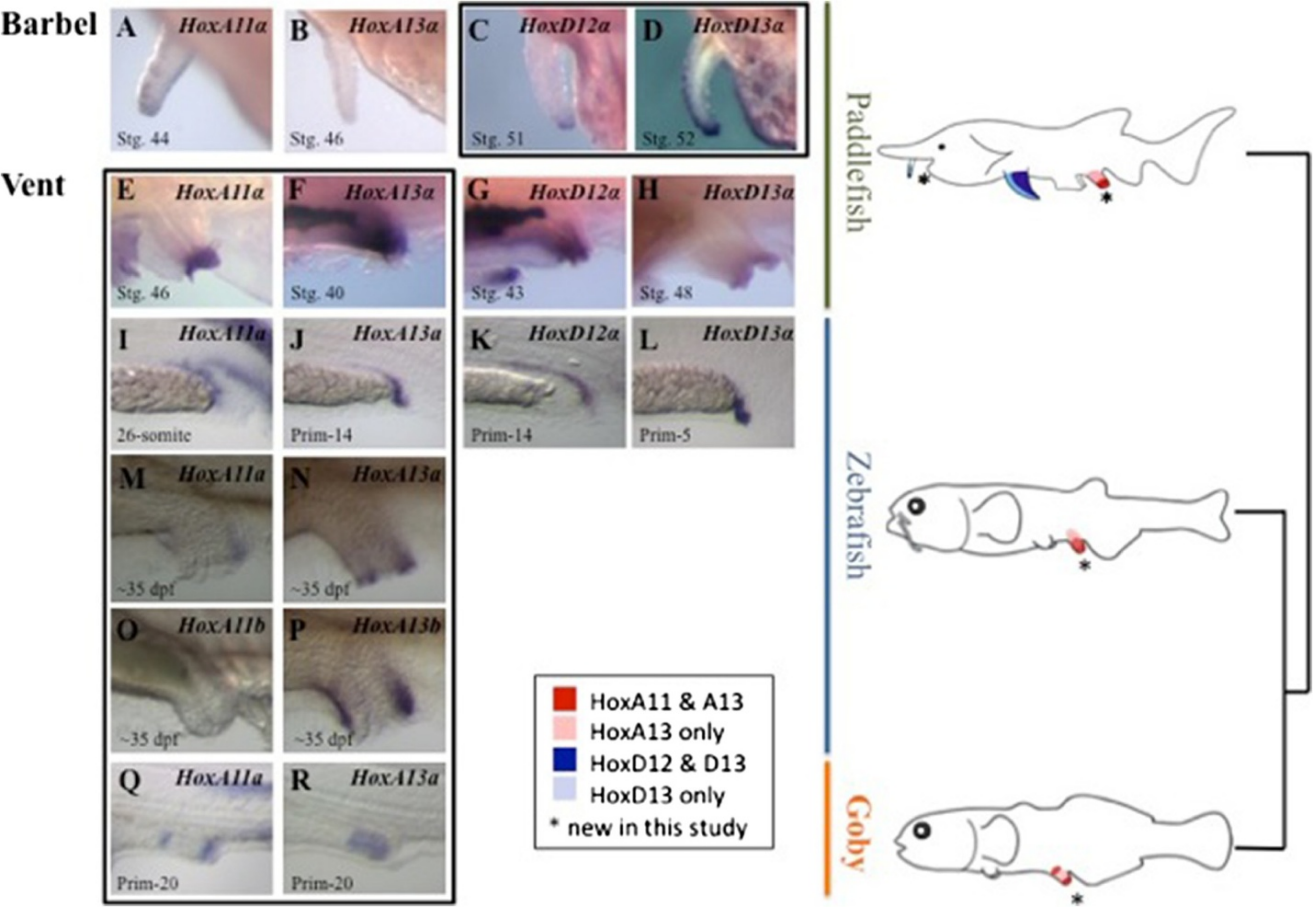
**The Hox distal phase/reverse collinear expression pattern is utilized by both the HoxD and HoxA clusters in a variety of distally elongated vertebrate structures.** The distal phase (DP) expression pattern occurs with the HoxD genes in the paddlefish barbel **(C-D)**, and with the HoxA genes in the vent of three ray-finned fishes **(E-F, I-J, M-R)**, outlined in black. *HoxD12* and *HoxD13* are expressed collinearly in the vent (**G-H, K-L**, not outlined in black), while *HoxA11*α, α, is expressed in the barbel **(A-B)**. The stage of peak expression for each gene is shown, but additional time series are shown in Additional files 4, 6-10. The phylogenetic relationships are indicated on the right, with cartoons of overlapping DP expression patterns. The distal phase HoxD expression pattern has previously been shown in the pectoral fins of paddlefish (shown in blue). However, we found that Hox DP expression is manifest with the posterior HoxA genes as well as the HoxD genes, and occurs in a variety of body plan features besides fins and limbs including the paddlefish barbel and vent of ray-finned fishes (shown in red). Teleost fishes (for example, zebrafish and goby) have two HoxA clusters; therefore, we feature expression of the HoxAa and Ab genes when both paralogs are present, and when expression occurs. Zebrafish has only one HoxD cluster, for which the expression patterns are shown. Gobies have two HoxD clusters, but only one *D12a* gene, and no *D13* genes. The paddlefish spiral valve is visible in **F-G**, and is black in color. In zebrafish, gut development occurs in association with the yolk sac extension (YSE), [38], a feature that is specific to zebrafish and their relatives, explaining the appearance of compressed expression domains in the hindgut region.

The paddlefish barbel is a sensory feature that develops in close proximity to the upper lip, has a cartilaginous core for support [see Additional file 4: Figure S3I] and is derived from the mandibular arch, or pharyngeal arch I [30]. During the final stages of barbel elongation, we found that *HoxA11* is expressed faintly at the distal margin and tip of the barbels (stg. 44, Figure 1A). We found no evidence for *HoxA13* expression in barbels based on three different approaches, which included full sequence transcriptome queries, paralog-specific quantitative-PCR analyses, and *in-situ* hybridization at multiple stages (Figure 1B, [see Additional file 4: Figure S3B], Table 1). The *HoxA13* probe used in the latter was validated via positive expression in pectoral fins, consistent with expected expression outlined by Davis *et al.*[11]. Therefore, this gene does not appear to play a role in patterning the paddlefish barbel.

*HoxD12* and *HoxD13* are expressed in the barbel, and they exhibit the DP expression pattern that has previously been described only in fins and limbs. At peak expression, *HoxD13* is expressed broadly in the barbel along the anterior margin and distal tip (stg. 52, Figure 1D), while *HoxD12* expression is restricted to a smaller domain at the distal tip (stg. 51, Figure 1C). These expression domains were consistent in multiple experiments with multiple probes that were paralog-specific. The broader expression of *HoxD13α*, relative to *HoxD12α*, was also supported by the relative number of hits in the transcriptome queries. Surprisingly, we found no expression for *HoxD13β* even though the transcriptome indicated it is expressed in the rostrum anlagen. Importantly, *HoxD13α* and *HoxD12α* occur at the 5′ end of the same Hox cluster [42]; therefore, the observed DP expression is consistent with coordinated regulation by shared cis-regulatory elements similar to what has been observed for DP HoxD expression in the tetrapod autopod [20] and pectoral fins of paddlefish and catshark [11, 12]. To determine whether HoxD genes undergo a switch from collinear to DP expression, as in the autopod/fin, we evaluated multiple developmental stages prior to and following this HoxD DP expression [see Additional file 4: Figure S3] and found no evidence for a switch from collinear to DP expression in the paddlefish barbel. While the barbel placode develops concurrently with early jaw patterning [30], Hox expression in the barbel does not occur until after jaw specification, which requires a Hox-free context for proper development [40, 41]. Therefore, Hox expression is not required for barbel elongation but may be associated with subsequent proximo-distal patterning. Regardless of its function in the barbel, this is the first report of any Hox expression in a derivative of the pharyngeal arch I.

Based on these findings in paddlefish, we used whole-mount *in situ* hybridization to investigate whether the posterior Hox genes are expressed in the developing barbels of zebrafish. Zebrafish have two pairs of barbels: the maxillary and nasal pairs. We found no evidence for expression of the focal Hox genes during development of zebrafish barbels [see Additional file 5: Figure S4], using probes that were validated in other structures and stages of development. These data suggest that there are multiple paths to patterning barbels in fishes, and provide support for independent origins of these structures. This is also indicated by morphological differences such as the absence of cartilage in zebrafish maxillary barbels [43].

### First evidence for reverse collinear expression of the HoxA genes

The DP expression pattern exhibited by the posterior HoxD genes has received much attention for its role in patterning distal regions of vertebrate appendages [9–12, 14, 20, 44], but to our knowledge there has been no report of DP expression for the posterior HoxA genes in any feature. Interestingly, it has been shown that the posterior HoxA genes share similar long range enhancer elements [45] and 5′ regulatory architecture as the HoxD genes, suggesting a shared origin in the ancestral Hox cluster [26].

Here, we report the first evidence of the DP pattern for the posterior HoxA genes in the hindgut and vent of three ray-finned fishes. The vent is a nonpaired, medial structure that protrudes from the body wall, is analogous to a urethra or cloaca, and exhibits morphological variation in different lineages. To validate the HoxA DP expression pattern in multiple taxa, we used a comparative approach to evaluate expression of the two posterior HoxA and HoxD genes in representatives of three ray-finned fishes during development of the hindgut and vent including a basal ray-finned fish (paddlefish, *Polyodon spathula*), a basal teleost (zebrafish, *Danio rerio*), and a derived teleost (blue-banded goby, *Lythrypnus dalli*). In the paddlefish vent, we found HoxA and HoxD expression; however, the DP pattern was manifest with the posterior HoxA genes only. During vent morphogenesis, *HoxA13* is expressed broadly throughout the paddlefish vent, while *HoxA11* is restricted to the most distal portion (Figures 1E-F and [see Additional file 6: Figure S5]), consistent with DP expression. The HoxA DP pattern in the vent of paddlefish was similar to the HoxD DP pattern observed in the paddlefish barbel in that there was no switch from an earlier collinear pattern.

Because this expression pattern in paddlefish represents the first documentation of DP expression for the HoxA genes, we felt it was important to investigate whether the HoxA DP expression pattern is conserved in other ray-finned fish lineages (that is, zebrafish and goby). While HoxA DP expression in the paddlefish vent occurred directly, we observed two waves of HoxA DP expression in the developing hindgut and vent of zebrafish, the first during embryonic patterning of the hindgut (approximately 30 hpf in zebrafish, Figure 1I-J and [see Additional file 7: Figure S6]) and the second wave much later (approximately 35 dpf) during larval morphogenesis of the vent and the emergence of pelvic fins, with the latter stage more comparable to the observed HoxA DP expression in the paddlefish vent (Figure 1M-P and [see Additional file 7: Figure S6]). Of the two duplicated HoxA clusters in zebrafish, the posterior HoxAa genes exhibit DP expression during both embryonic hindgut and larval vent patterning (Figure 1I-J, M-N), while only one HoxAb gene was expressed during the later stage of vent patterning. We found broad *HoxA13b* expression in the larval vent, with no expression of *HoxA11b* (Figure 1O-P and [see Additional file 7: Figure S6]), which might be consistent with 5′ regulation of DP expression, but remains indeterminate based on expression of a single gene.

In the goby, HoxA DP expression is observed during the early stages of embryonic hindgut patterning and is more complex than zebrafish. Instead of an initial HoxA DP expression pattern, as in the paddlefish and zebrafish hindgut and vent, we observed a switch from broad *HoxA11a* expression to a more restricted expression domain relative to *HoxA13a* (Figure 1Q-R and [see Additional file 8: Figure S7]), which might be consistent with a switch from collinear to DP expression. We did not observe expression of the posterior HoxAb genes in the goby, at any stage. Additionally, we did not observe expression of HoxA genes at later stages during the morphogenesis of the vent or cloaca/genital papilla (which are separate structures in this species) in the larvae, which may be associated with morphological and functional disparity of the goby vent/genital papilla relative to the zebrafish and paddlefish vent.

While the HoxA DP expression pattern occurs directly in paddlefish, we have also demonstrated the DP pattern in at least one paralogous HoxA cluster (where applicable), at multiple stages of development, in two additional lineages ray-finned fishes, where *HoxA13* is expressed more broadly than *HoxA11*-a hallmark the DP expression pattern.

HoxA genes are known to be expressed during hindgut development in tetrapods and cartilaginous fishes. *HoxA13* is expressed in the hindgut of the little skate, and the HoxA genes are expressed in a collinear pattern in the developing gut of chick and mouse [46–48]. However, these data are the first documentation of the DP HoxA expression pattern. Therefore, HoxA DP expression does not appear to occur in the hindgut of tetrapods and remains to be demonstrated in ancestral jawed vertebrates because comparative patterns of HoxA expression have not been characterized in cartilaginous fishes.

HoxD genes are expressed in the hindgut/vent of ray-finned fishes, but do not exhibit the DP pattern. The posterior HoxD genes have been shown to exhibit collinear expression during development of urogenital structures in several vertebrates. For example, *HoxD12* and *HoxD13* are expressed in the mammalian genital bud [15, 49], the cloaca of the catshark, and may play a role in the morphogenesis of catshark claspers [12]. Further, these genes are expressed in the hindgut of zebrafish, but only *HoxD13* was found to be expressed in the zebrafish vent [50]. We find similar results in zebrafish for *HoxD12* and *HoxD13* (Figure 1K-L and [see Additional file 9: Figure S8]), and show a similar pattern in the paddlefish hindgut/vent, where *HoxD12* is expressed broadly in the vent, while expression of *HoxD13* is restricted to the distal tip (Figure 1G-H and [see Additional file 10: Figure S9]).

### Temporal mode of Hox expression varies between species

The HoxD DP expression pattern has been referred to as ‘biphasic’ [12], ‘late phase’ and ‘autopodial-like’ [11]. We clarify that DP patterns are not restricted to fins and limbs, nor to the HoxD genes, and are not necessarily associated with a switch to a ‘late phase’ of expression. For example, the HoxD DP pattern occurs directly in paddlefish barbels, with no earlier phase of expression [see Additional file 4: Figure S3], consistent with the notion that regulation of the DP pattern is distinct from early, proximal, and collinear expression patterns. [13]. Notably, collinear and DP expression occurred in concert in paddlefish structures with collinear HoxD expression, co-occurring in vent and early pectoral fin at stages 39 to 46), and with DP HoxD expression co-occurring in the barbel and later stages of pectoral fin development (at stages 51 to 52). Finally, pelvic and pectoral fin development, along with their associated Hox expression patterns, occurs in closer successive stages in paddlefish, but is disjunct and protracted in zebrafish and goby.

HoxA DP expression occurs early in zebrafish and goby hindgut (prim 14, approximately 30 hpf in zebrafish) in concert with primary axial expression and well before the emergence of the fins and their associated Hox expression patterns. But in the vent, HoxA DP expression occurs much later in zebrafish (35 dpf), coincident with pectoral fin morphogenesis and pelvic fin emergence. Overall, it appears that HoxA DP expression in paddlefish vent and zebrafish hindgut and vent are not associated with a switch from earlier collinear expression. Rather, the HoxA DP pattern is established and re-established at different times during development and patterning of distally elongated structures. Descriptions of ‘early/late phase’ expression, or similar terminology, may be useful with respect to focal structures but can be confounding with respect to overall waves of Hox expression in the body plan or in features that have not been previously evaluated.

### The Hox DP expression pattern appears to be an ancient module that is associated with multiple Hox clusters and the development of a variety of structures

Recent work has focused on understanding the regulatory mechanisms associated with HoxD DP expression in the developing fins and limbs of vertebrates [5, 8, 26]. The HoxD cluster is flanked by two topological domains: the 3′ telomeric and the 5′ centromeric regulatory regions. There is a switch in the regulation of the posterior HoxD genes (9 to 11) from the telomeric to the centromeric regulatory domain, which is associated with expression in the forelimb and the distal autopod, respectively, in mice [5]. In addition, DP HoxD expression occurs in distal structures of paired fins of paddlefish and sharks, and in zebrafish the regulatory regions map to the same 5′ gene desert that is conserved in vertebrates [12, 17].

Although HoxA DP expression has not been observed in fins or limbs, it is predicted by similarities in the 5′ regulatory landscapes of the HoxA and HoxD clusters in jawed vertebrates. However, it remains to be seen whether the upstream (5′) regulatory regions on the HoxA cluster can be functionally linked with DP expression patterns in hindgut and vent, through reporter gene constructs and deletion experiments. In the mouse autopod, HoxA DP expression may be masked by a dominant negative interaction between *HoxA13* and *HoxA11*[21, 26]. The lack of HoxA DP expression has remained elusive in zebrafish because it occurs in a medial structure at stages of development not previously scrutinized. Here, we demonstrate a reverse collinear expression pattern of posterior HoxA genes in the developing embryonic hindgut and larval vent in zebrafish, which is consistent with independent regulation by enhancers in the 5′ upstream region of the HoxA cluster. The HoxA cluster is in a different orientation than the HoxD cluster; therefore, we use 5′ rather than centromeric, see [26]. Finally, it has been suggested that developing paired appendages and hindgut are associated in more ways than shared Hox expression patterns. Several key innovations following the two rounds of genome duplications in the stem lineage of vertebrates allowed for both the regionalization of the gastrointestinal tract and the emergence of paired appendages from the somatopleure in jawed vertebrates [51–53]. We demonstrate that HoxA DP expression is utilized to differentially pattern the distal gut, in a fashion that is reminiscent of what has been observed in the distal region of paired appendages.

We note that HoxD and HoxA DP expression in the paddlefish barbel and ray-finned fish hindgut/vent meets the criteria described for ‘autopodial’ expression (sensu [13]), with the exception that it does not occur while bony elements are being specified (as in digits). The paddlefish barbel does have a cartilaginous core, which stains with Alcian blue, but Hox expression occurs after it develops, likely due to concurrent jaw development. The vent in paddlefish and zebrafish does not exhibit cartilage or bony elements at the stages we evaluated. Therefore, DP Hox expression does not appear to be restricted to specifying identity of bony elements. However, both barbels and vents exhibit proximo-distal axes, and we propose that the definition of DP expression should include proximal expansion of the 5′gene, in addition to or in lieu of, anterior expansion.

## Conclusions

While the genetic underpinnings of the fin to limb transition are clearly a spectacular example of evolution, this historical bias may have diverted attention from other aspects of Hox gene evolution, and their role in morphological diversity. By investigating a broader range of developmental stages and novel structures in a variety of lineages, we demonstrate that Hox genes are expressed in an anterior domain (in a structure with a proximo-distal axis) that is a derivative of pharyngeal arch I (the paddlefish barbel), which has not been previously documented in any taxon. Further, this HoxD expression pattern is similar to reverse collinear expression (which we call DP), which was previously thought to be associated only with the development of paired appendages in vertebrates. Finally, we demonstrate that the Hox DP expression pattern is also a feature of the posterior HoxA genes, indicating that the DP pattern of Hox expression is not restricted to appendages or the HoxD genes, nor is it associated with any particular novelty. Rather, these findings suggest that the regulatory domains associated with DP expression existed in the ancestral Hox cluster before duplication in the stem lineage of jawed vertebrates. Therefore, Hox DP expression may be an ancestral feature of the Hox regulatory network that has been co-opted to pattern a wide range of body plan features by the HoxA and HoxD clusters. These findings provide a new line of evidence supporting the redeployment of preexisting patterning programs and broadly implicate Hox DP expression in the evolution of morphological diversity, suggesting deep homology of distally elongated structures in vertebrates.

## Electronic supplementary material

Additional file 1: Figure S1: Evidence for *HoxA11* alpha expression in the paddlefish rostrum transcriptome. The known paddlefish *HoxA11* alpha and beta sequences were used as the queries for searching the paddlefish rostrum transcriptome database. This figure illustrates the distribution of 23 blast hits to *HoxA11* alpha. The first 89 nucleotides of the paddlefish sequence was used for the BLASTN. Results, as shown, indicate significant sequence similarity for *HoxA11* in several fishes and other vertebrates. A contig alignment of the first 125 nucleotides from the paddlefish rostrum transcriptome database and other regions spanning variable sites in the *HoxA11* paralogs (indicated in orange) are shown. None of the blast hit sequences were unique to the *HoxA11* beta sequence, but several contained unique sequences in the *HoxA11* alpha sequence. For example, a 60-bp contig spanning the exon-exon boundary was identical to the *HoxA11* alpha sequence, indicating clear evidence for *HoxA11α* expression in the anterior region of the paddlefish. (PNG 80 KB)

Additional file 2: Figure S2: Cartoon of probes constructed for posterior HoxA and HoxD genes from three ray-finned fishes. (PNG 374 KB)

Additional file 3: Table S1: Primers used to amplify DNA with PCR for *in situ* hybridization probes. (PNG 160 KB)

Additional file 4: Figure S3: Hox expression in paddlefish barbels. *HoxA11α* is expressed at the distal tip of the barbel at stage 44 and turns off by stage 52 (A). *HoxA13α* was not detected in barbels at any stage examined (B). *HoxD12α* is not expressed at stage 46. Peak expression of *HoxD12α* is at stage 51, and is restricted to the distal tip of the barbel (C). *HoxD13α* is also not expressed at stage 46. *HoxD13α* expression appears by stage 51, and is concentrated to the distal tip of the barbel, though slightly broader than *HoxD12α* expression (D). *HoxD13α* expression expands through stage 52, and exhibits the broadest expression domain of all genes tested (E,G). Expression of *HoxD13α* wanes in the barbel after stage 52 (F,H). Anterior to the left, dorsal up in A-F. Anterior left, right lateral up in the ventral views in G-H. Barbel at stg.53 with Alcian blue staining indicating the cartilaginous core (I). (PNG 383 KB)

Additional file 5: Figure S4: Hox genes do not pattern the barbels of zebrafish. We found no evidence of Hox expression in the developing maxillary or nasal barbels of zebrafish using whole mount *in situ* hybridization. Expression of the posterior *HoxAa* (A), *HoxAb* (B), and *HoxDa* (C) genes were examined from the initiation of barbel growth (just following the appearance of pelvic rays) through post juvenile stages (9.2 to 13.0 SSL). Pictures shown are of juvenile stages, approximately 11.0 SSL. Maxillary barbels are marked with an asterisk (*) and nasal barbels with an arrow. Photos were taken from a dorso-lateral view of the left barbels; anterior is up and left lateral is to the left. (PNG 389 KB)

Additional file 6: Figure S5: Distal phase expression of *HoxA11* *α* and *HoxA13* *α* during vent differentiation in paddlefish. Developmental series of paddlefish vent morphogenesis showing *HoxA11α* and *HoxA13α* expression. Peak expression of *HoxA11α* occurs during stage 46, and is concentrated at the distal margin of the vent (A). *HoxA13α* expression starts earlier, during stages 40 to 44, and remains broader than *HoxA11α* expression (B). Therefore, the HoxA genes display reverse collinearity in the developing vent of paddlefish. *HoxA13α* expression along the cylindrical circumference of the vent is clearly seen in from a ventral view (C). Anterior to the left, dorsal up in A-B. Anterior to the left, left lateral up in C. (PNG 218 KB)

Additional file 7: Figure S6: Expression of posterior HoxA genes in the developing hindgut and vent of embryonic and larval zebrafish. The expression domains of two genes from each of the *HoxAa* and *HoxAb* clusters were examined in order to look for nested and overlapping patterns of expression. Reverse collinearity is seen in the HoxAa genes (A-B), whereas the HoxAb genes display a collinear expression pattern (C-D). Black arrowheads mark the furthest anterior expression of each gene at any time point. Expression domains of these genes were also examined in the vents of larval zebrafish, approximately 30–38 dpf (A-D: 6.3 SSL and 7.2 SSL). Larval fish were sampled from the time when the anal and dorsal fin structures were emerging through barbel development (6.3 SSL – 13.0 SSL); however, vent expression was only observed early within this period, through development of the pelvic fins (6.3 – 8.3 SSL). The expression patterns of both the HoxAa and HoxAb genes are consistent with DP expression, where the *HoxA13* gene is expressed more broadly than the *HoxA11* gene. Anterior is to the left and dorsal is up in all photos. (PNG 207 KB)

Additional file 8: Figure S7: Expression of the posterior HoxA genes in the developing hindgut and vent of the embryonic and larval blue-banded goby. Embryonic and larval stages of the blue-banded goby were sampled and stained for Hox expression using *in situ* hybridization. HoxAa expression displays a switch in patterns from the 14-somite stage, when *HoxA11a* is widely expressed in the posterior gut (A) and *HoxA13a* is expressed faintly in a small portion of the posterior gut (B, arrowhead). By prim-23, *HoxA13a* is expressed broadly in the posterior gut, whereas *HoxA11a* expression is limited to two domains on either side of the *HoxA13a* expression. We found no expression of *HoxA11b* and *HoxA13b* at any stage examined (C-D). Expression of these four genes were also examined in the vents of larval gobies (A-D, 5.2 SSL). The larvae were sampled at stages from the condensation of the dorsal and anal fins through the development of the pelvic fins (4.0 to 8.9 SSL); however, there was no reappearance of Hox gene expression. Gobies have two HoxD clusters, but only one *HoxD12a* gene, and no *HoxD13* genes; therefore, we did not examine these genes for collinear patterns. Anterior is to the left and dorsal is up in all photos. (PNG 328 KB)

Additional file 9: Figure S8: Expression of posterior HoxD genes in the developing hindgut and vent of embryonic and larval zebrafish. Zebrafish has one HoxD cluster, and therefore does not have *HoxD12b* or *HoxD13b* genes. The *HoxDa* genes are expressed in a collinear pattern in zebrafish embryos (A-B). Black arrowheads mark the furthest anterior expression for each gene at any time point. Expression domains of these two genes were also examined in the vents of larval zebrafish approximately 30 to 38 dpf (A-B: 6.3 SSL and 7.2 SSL). Larval fish were sampled from when the anal and dorsal fin structures were emerging through barbel development (6.3 SSL to 13.0 SSL). No expression of the HoxD cluster was observed during these later stages of development. Anterior is to the left and dorsal is up in all photos. (PNG 207 KB)

Additional file 10: Figure S9: Collinear expression of *HoxD12α* and *HoxD13α* during vent differentiation in paddlefish. Developmental series showing the peak of *HoxD12α* and *HoxD13α* expression domains. *HoxD12α* expression peaks at stage 43, when it surrounds the distal margin of the vent and lines the cylindrical circumference of the hindgut (A). Peak of expression of *HoxD13α* occurs at stage 48, when it is limited to the distal-most margin of the vent (B). Anterior is to the left, dorsal up in A-B. Ventral view of stage 46 and 48 for *HoxD13α*, showing expression in the cylindrical circumference of the vent (C). Anterior is to the left and left lateral is up in C. (PNG 328 KB)

## Abbreviations

DP: distal phase
YSE: yolk sac extension.
