# Supplementary figures and images for: HoxA and HoxD expression in a variety of vertebrate body plan features reveals an ancient origin for the distal Hox program
Source: EvoDevo. 2014 Nov 19;5:44. doi: 10.1186/2041-9139-5-44 (PMC4407844; doi:10.1186/2041-9139-5-44)

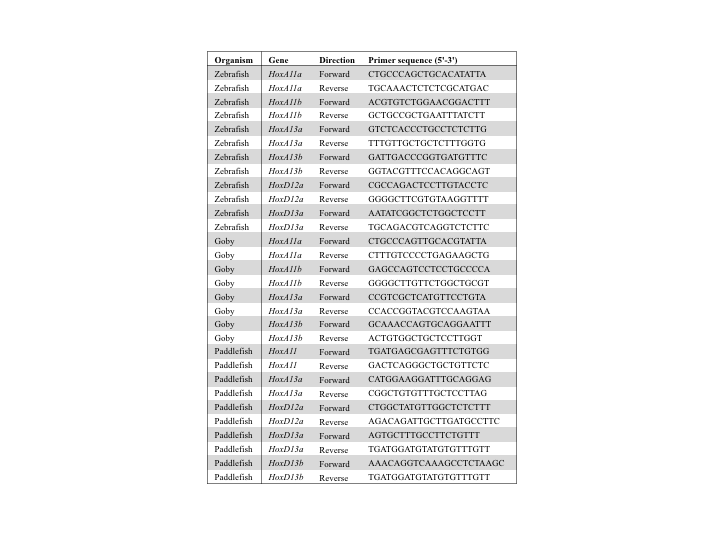

Supplement: Supplementary file 1 — Additional file 1: Figure S1: Evidence for HoxA11 alpha expression in the paddlefish rostrum transcriptome. The known paddlefish HoxA11 alpha and beta sequences were used as the queries for searching the paddlefish rostrum transcriptome database. This figure illustrates the distribution of 23 blast hits to HoxA11 alpha. The first 89 nucleotides of the paddlefish sequence was used for the BLASTN. Results, as shown, indicate significant sequence similarity for HoxA11 in several fishes and other vertebrates. A contig alignment of the first 125 nucleotides from the paddlefish rostrum transcriptome database and other regions spanning variable sites in the HoxA11 paralogs (indicated in orange) are shown. None of the blast hit sequences were unique to the HoxA11 beta sequence, but several contained unique sequences in the HoxA11 alpha sequence. For example, a 60-bp contig spanning the exon-exon boundary was identical to the HoxA11 alpha sequence, indicating clear evidence for HoxA11α expression in the anterior region of the paddlefish. (PNG 80 KB) [file 13227_2014_137_MOESM1_ESM.png]

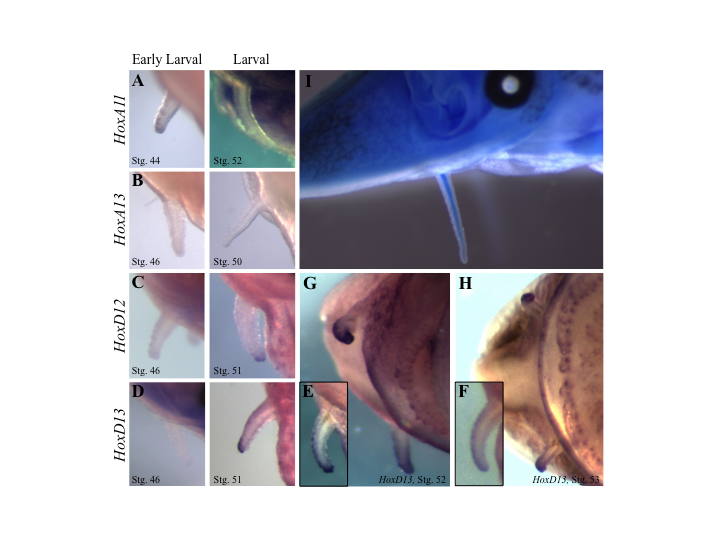

Supplement: Supplementary file 2 — Additional file 2: Figure S2: Cartoon of probes constructed for posterior HoxA and HoxD genes from three ray-finned fishes. (PNG 374 KB) [file 13227_2014_137_MOESM2_ESM.png]

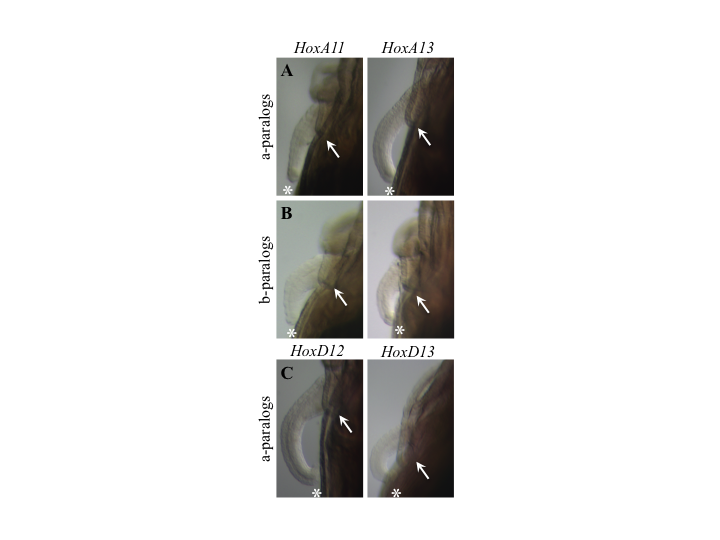

Supplement: Supplementary file 3 — Additional file 3: Table S1: Primers used to amplify DNA with PCR for in situ hybridization probes. (PNG 160 KB) [file 13227_2014_137_MOESM3_ESM.png]

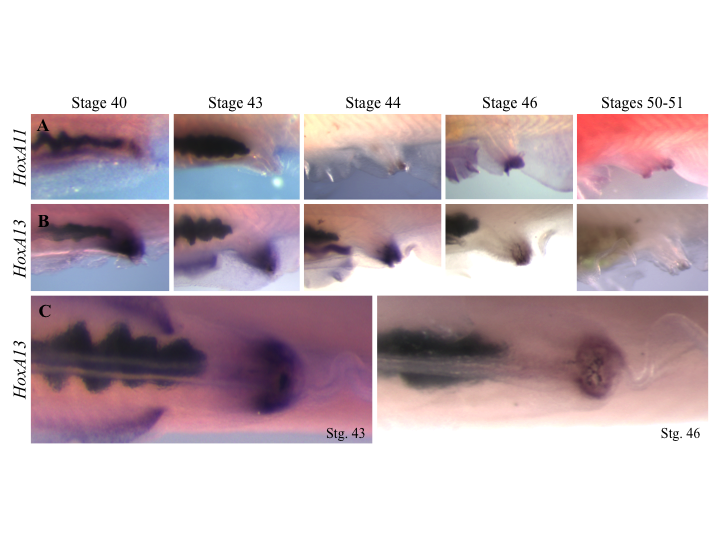

Supplement: Supplementary file 4 — Additional file 4: Figure S3: Hox expression in paddlefish barbels. HoxA11α is expressed at the distal tip of the barbel at stage 44 and turns off by stage 52 (A). HoxA13α was not detected in barbels at any stage examined (B). HoxD12α is not expressed at stage 46. Peak expression of HoxD12α is at stage 51, and is restricted to the distal tip of the barbel (C). HoxD13α is also not expressed at stage 46. HoxD13α expression appears by stage 51, and is concentrated to the distal tip of the barbel, though slightly broader than HoxD12α expression (D). HoxD13α expression expands through stage 52, and exhibits the broadest expression domain of all genes tested (E,G). Expression of HoxD13α wanes in the barbel after stage 52 (F,H). Anterior to the left, dorsal up in A-F. Anterior left, right lateral up in the ventral views in G-H. Barbel at stg.53 with Alcian blue staining indicating the cartilaginous core (I). (PNG 383 KB) [file 13227_2014_137_MOESM4_ESM.png]

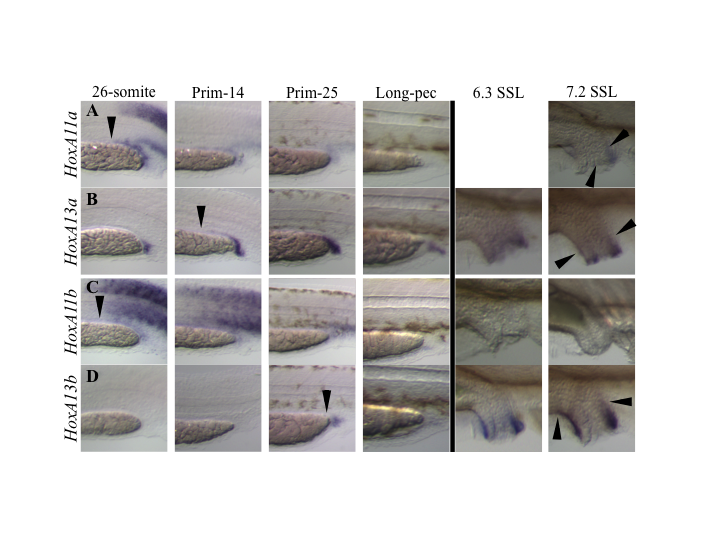

Supplement: Supplementary file 5 — Additional file 5: Figure S4: Hox genes do not pattern the barbels of zebrafish. We found no evidence of Hox expression in the developing maxillary or nasal barbels of zebrafish using whole mount in situ hybridization. Expression of the posterior HoxAa (A), HoxAb (B), and HoxDa (C) genes were examined from the initiation of barbel growth (just following the appearance of pelvic rays) through post juvenile stages (9.2 to 13.0 SSL). Pictures shown are of juvenile stages, approximately 11.0 SSL. Maxillary barbels are marked with an asterisk (*) and nasal barbels with an arrow. Photos were taken from a dorso-lateral view of the left barbels; anterior is up and left lateral is to the left. (PNG 389 KB) [file 13227_2014_137_MOESM5_ESM.png]

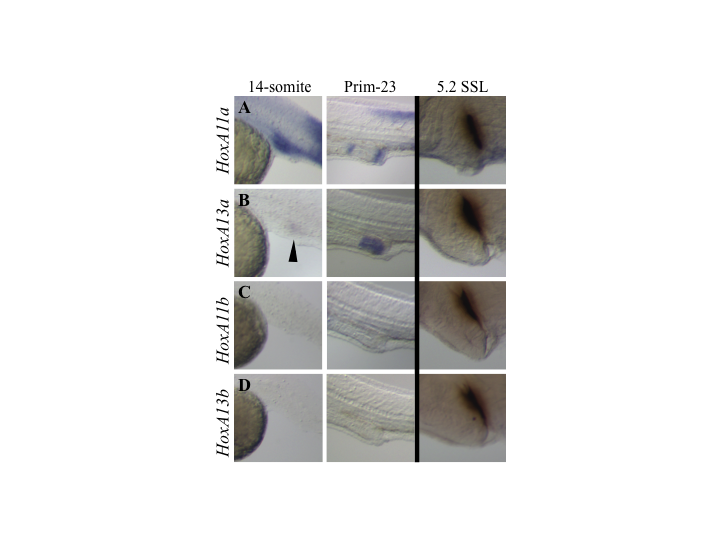

Supplement: Supplementary file 6 — Additional file 6: Figure S5: Distal phase expression of HoxA11 α and HoxA13 α during vent differentiation in paddlefish. Developmental series of paddlefish vent morphogenesis showing HoxA11α and HoxA13α expression. Peak expression of HoxA11α occurs during stage 46, and is concentrated at the distal margin of the vent (A). HoxA13α expression starts earlier, during stages 40 to 44, and remains broader than HoxA11α expression (B). Therefore, the HoxA genes display reverse collinearity in the developing vent of paddlefish. HoxA13α expression along the cylindrical circumference of the vent is clearly seen in from a ventral view (C). Anterior to the left, dorsal up in A-B. Anterior to the left, left lateral up in C. (PNG 218 KB) [file 13227_2014_137_MOESM6_ESM.png]

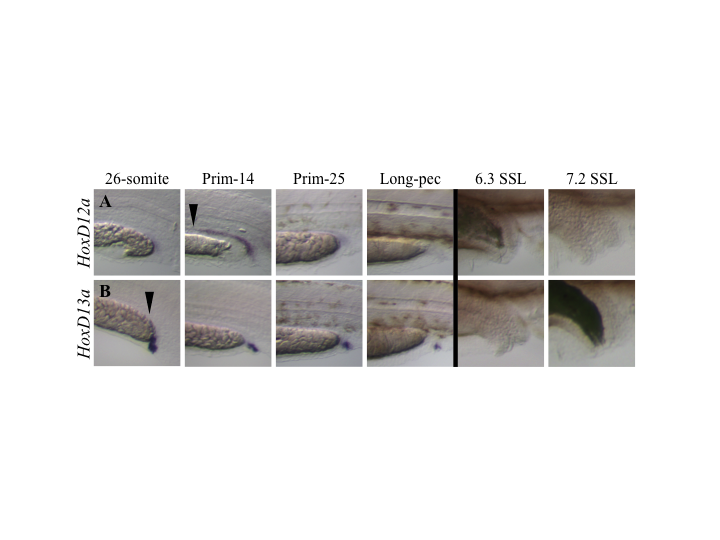

Supplement: Supplementary file 7 — Additional file 7: Figure S6: Expression of posterior HoxA genes in the developing hindgut and vent of embryonic and larval zebrafish. The expression domains of two genes from each of the HoxAa and HoxAb clusters were examined in order to look for nested and overlapping patterns of expression. Reverse collinearity is seen in the HoxAa genes (A-B), whereas the HoxAb genes display a collinear expression pattern (C-D). Black arrowheads mark the furthest anterior expression of each gene at any time point. Expression domains of these genes were also examined in the vents of larval zebrafish, approximately 30–38 dpf (A-D: 6.3 SSL and 7.2 SSL). Larval fish were sampled from the time when the anal and dorsal fin structures were emerging through barbel development (6.3 SSL – 13.0 SSL); however, vent expression was only observed early within this period, through development of the pelvic fins (6.3 – 8.3 SSL). The expression patterns of both the HoxAa and HoxAb genes are consistent with DP expression, where the HoxA13 gene is expressed more broadly than the HoxA11 gene. Anterior is to the left and dorsal is up in all photos. (PNG 207 KB) [file 13227_2014_137_MOESM7_ESM.png]

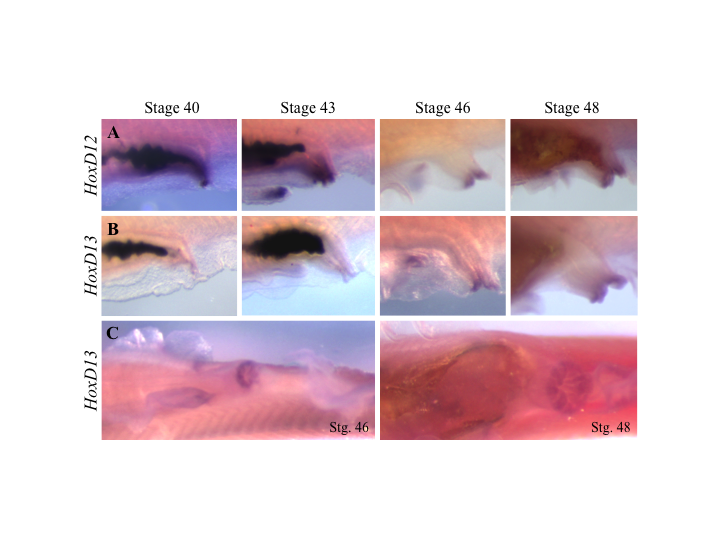

Supplement: Supplementary file 8 — Additional file 8: Figure S7: Expression of the posterior HoxA genes in the developing hindgut and vent of the embryonic and larval blue-banded goby. Embryonic and larval stages of the blue-banded goby were sampled and stained for Hox expression using in situ hybridization. HoxAa expression displays a switch in patterns from the 14-somite stage, when HoxA11a is widely expressed in the posterior gut (A) and HoxA13a is expressed faintly in a small portion of the posterior gut (B, arrowhead). By prim-23, HoxA13a is expressed broadly in the posterior gut, whereas HoxA11a expression is limited to two domains on either side of the HoxA13a expression. We found no expression of HoxA11b and HoxA13b at any stage examined (C-D). Expression of these four genes were also examined in the vents of larval gobies (A-D, 5.2 SSL). The larvae were sampled at stages from the condensation of the dorsal and anal fins through the development of the pelvic fins (4.0 to 8.9 SSL); however, there was no reappearance of Hox gene expression. Gobies have two HoxD clusters, but only one HoxD12a gene, and no HoxD13 genes; therefore, we did not examine these genes for collinear patterns. Anterior is to the left and dorsal is up in all photos. (PNG 328 KB) [file 13227_2014_137_MOESM8_ESM.png]
